# Analgesia after major laparoscopic surgery in patients with chronic kidney disease: A retrospective cohort study

**DOI:** 10.1038/s41598-019-40627-1

**Published:** 2019-03-08

**Authors:** Hey-ran Choi, Tak Kyu Oh, Jinhee Kim, Young-Tae Jeon

**Affiliations:** 1 Department of Anesthesiology and Pain Medicine, Inje University Seoul Paik Hospital, Seoul, South Korea; 2 Department of Anesthesiology and Pain Medicine, Seoul National University Bundang Hospital, Seoul, South Korea

## Abstract

The amount of reduction in opioid doses and its effect on postoperative pain outcomes in chronic kidney disease (CKD) patients in the perioperative setting remains unclear. This study aimed to investigate differences in postoperative pain outcomes after major laparoscopic surgery between patients with CKD and those with normal preoperative kidney function. Medical records of patients who underwent laparoscopic major abdominal surgery from January 2010 to December 2016 were retrospectively reviewed, and 6,612 patients were finally included. During postoperative day (POD) 0–3, patients with an estimated glomerular filtration rate (eGFR) < 30 mL min^−1^ 1.73 m^−2^ had 3.5% lower morphine equivalent consumption than those with an eGFR ≥ 90 mL min^−1^ 1.73 m^−2^ (*P* = 0.023), whereas patients with preoperative eGFR between 60–90 mL min^−1^ 1.73 m^−2^ and 30–60 mL min^−1^ 1.73 m^−2^ showed no significant differences in morphine equivalent consumption. Additionally, pain scores at rest during POD 0–3 were not significantly associated with preoperative kidney function. In conclusion, our results suggest that patients with mild to moderate CKD (stage 2–3) did not require reduction of opioid analgesics during POD 0–3, compared to patients with normal preoperative kidney function. Only patients with severe CKD (stage ≥ 4) might require a slight reduction of opioid analgesics.

## Introduction

It is reported that about 80% of all surgical patients experience postoperative pain^1^, and effective pain control plays an important role in lowering postoperative morbidity and mortality^2^. The most commonly used method of postoperative pain control is systemic opioid analgesia^3^. A recent study reported that opioids are commonly used until postoperative days (PODs) 4 to 15 among patients after common surgical procedures^4^. However, opioids are associated with a variety of known adverse effects^5^ and should be used with caution in patients who are more vulnerable to these adverse effects. Patients with chronic kidney disease (CKD) are particularly at risk of developing serious adverse effects of opioids.

CKD, which is defined by an estimated glomerular filtration rate (eGFR) below 60 mL min^−1^ 1.73 m^−2^ ^6^, is a global health problem with a worldwide prevalence of 8% to 16%^7^. Because CKD patients have a reduced ability to clear drugs through the kidney, the dose of opioids should be appropriately reduced to avoid adverse effects in these patients^8^. A systematic review conducted as part of the European Palliative Care Research Collaborative opioid guidelines project^9^ reported that, among opioids, morphine has the highest toxicity rate in CKD patients, whereas alfentanil, fentanyl, and methadone have the lowest toxicity rates. However, that study only analysed data from chronic cancer patients and did not include patients with perioperative or acute pain^9^. Although one study recommended that the dose of transdermal fentanyl should be reduced by 50% in CKD patients^10^, there is limited evidence regarding the use of fentanyl for postoperative and acute pain control in patients with CKD^11^. Thus, there is a lack of information regarding the impact of impaired renal function in patients with CKD on requirement for opioids and on pain outcomes in the perioperative setting.

Therefore, this study aimed to investigate differences in postoperative pain outcomes, including opioid consumption and pain scores, after major laparoscopic surgery, between patients with CKD and those with normal kidney function. Our hypothesis for this study was that a correlation exists between the degree of renal impairment and opioid consumption in patients with CKD stages 1–5, after major laparoscopic surgery

## Results

**Figure 1:**
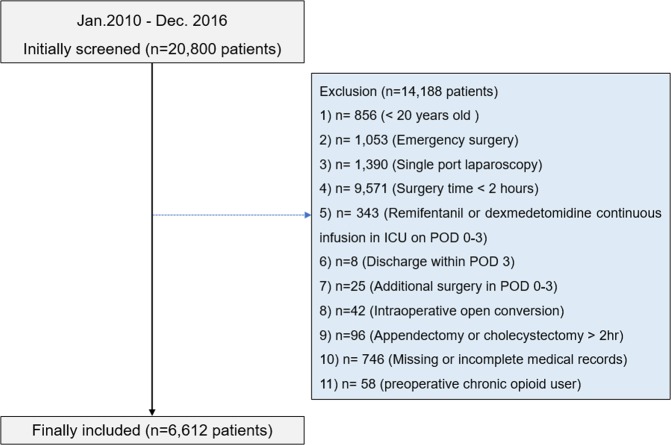
Patient selection flowchart.

A total of 20,800 patients at the Seoul National University Bundang Hospital (SNUBH) underwent a laparoscopic surgical procedure between January 1, 2010 and December 2016. Of these, 856 patients were excluded for age younger than 20 years, 1,053 were excluded for emergency surgery, 1,390 were excluded for single-port laparoscopy, 9,571 were excluded for surgery lasting less than 2 hours, 343 were excluded for receiving a continuous infusion of remifentanil or dexmedetomidine during the first three PODs, 8 were excluded for being discharged before the third POD, 25 were excluded for undergoing additional surgery during the first three PODs, 42 were excluded for intraoperative conversion to open laparotomy, 96 were excluded for undergoing simple appendectomy or cholecystectomy, 58 were excluded due to preoperative chronic opioid usage, and 746 were excluded for missing or incomplete medical records. As a result, 6,612 patients were included in the final analysis. A flowchart of the patient selection process is shown in Fig. 1 and characteristics of the included patients are shown in Table 1. The mean (standard deviation [SD]) preoperative eGFR was 96.9 mL min^−1^ 1.73 m^−2^ (25.8). There were no life-threatening opioid-related complications (e.g., respiratory arrest) among the study population.

**Table 1:** Characteristics of patients who underwent major laparoscopic abdominal surgery from 2010–2016.

Variables	Total (n = 6,612)	Mean (SD)
Age (years)		58.7 (13.2)
Body mass index (kg m^−2^)		24.1 (3.4)
Sex: male	3,715 (56.2%)	
**Preoperative comorbidities**		
ASA class		
1	2,839 (42.9%)	
2	3,542 (53.6%)	
≥3	231 (3.5%)	
Hypertension	1,930 (29.2%)	
Diabetes mellitus	948 (14.3%)	
Ischemic heart disease	288 (4.4%)	
Cerebrovascular disease	169 (2.6%)	
Liver disease	192 (2.9%)	
Cancer	5,054 (76.4%)	
eGFR^a^, mL min^−1^ 1.73 m^−2^		96.9 (25.8)
eGFR: ≥90	1,382 (20.9%)	
eGFR: <90	2,615 (39.5%)	
eGFR: 60–90	2,330 (35.2%)	
eGFR: 30–60	248 (3.8%)	
eGFR < 30	37 (0.6%)	
**Information regarding surgical procedures**		
Surgery time, min	196.4 (68.4)	
Propofol based intravenous anaesthesia	1,188 (18.0%)	
Intraoperative magnesium sulphate infusion	704 (10.6%)	
Length of hospital stay (days) after surgery	6.9 (6.3)	
**Years of surgery**		
2010–2012	2,205 (33.3%)	
2013–2014	1,770 (26.8%)	
2015–2016	2,637 (39.9%)	
**Postoperative analgesics consumption on POD 0–3**		
Oral morphine equivalent consumption, mg		516.6 (303.3)
Acetaminophen, mg		341.2 (812.9)
Ketorolac, mg		47.8 (230.9)
**Numeric rating scale, pain score at rest**		
POD 0		5.4 (1.6)
POD 1		4.5 (1.1)
POD 2		3.6 (1.0)
POD 3		3.1 (1.0)

Preoperative eGFR^a^ (mL min^−1^ 1.73 m^−2^): 186 × (Creatinine)^−1.154^ × (Age)^−0.203^ × (0.742, if female).

SD, standard deviation; ASA, American Society of Anesthesiologists; POD, postoperative day; eGFR, estimated glomerular filtration rate.

### Morphine equivalent consumption during the first three PODs

Table 2 shows the results of the univariate generalized linear regression analysis of morphine equivalent consumption on PODs 0 to 3. Sex, body mass index (BMI, kg m^−2^), American Society of Anesthesiologists (ASA) physical status, some preoperative comorbidities (hypertension, diabetes mellitus, cancer), surgery time, propofol-based total intravenous anaesthesia (TIVA), intraoperative magnesium infusion, years of surgery, preoperative eGFR were selected with a criterion of *P* < 0.2 in the univariate model. The results of the multivariate generalized linear regression analysis, including the variables selected from the univariate model, are shown in Table 3. In the multivariate model, the group with a preoperative eGFR < 30 mL min^−1^ 1.73 m^−2^ had a significant 3.5% lower morphine equivalent consumption on PODs 0 to 3 than the group with a preoperative eGFR ≥ 90 mL min^−1^ 1.73 m^−2^ (exponentiated [exp] regression coefficient: −0.035, 95% confidence interval [CI], −0.064, −0.005; *P* = 0.023). However, the patients with preoperative eGFR: 60–90 mL min^−1^ 1.73 m^−2^ and 30–60 mL min^−1^ 1.73 m^−2^ were not associated with differences in morphine equivalent consumption on POD 0–3 (*P* = 0.145 and *P* = 0.222, respectively).

**Table 2:** Univariate generalized linear regression analysis for morphine equivalent (mg) consumption on postoperative day 0–3 after major laparoscopic abdominal surgery from 2010–2016.

Characteristics		Exp. Coefficient (95% CI)	*P*-value
Age, years		−0.001 (−0.002, 0.001)	0.999
Sex: Male (vs Female)		0.385 (0.358, 0.413)	<0.001
Body Mass Index, kg m^−2^		0.019 (0.015, 0.023)	<0.001
ASA	2 (vs 1)	0.021 (−0.055, 0.097)	0.596
	3 (vs 1)	0.059 (0.030, 0.087)	<0.001
**Preoperative Comorbidity**			
	Hypertension	0.043 (0.013, 0.073)	0.005
	Diabetes mellitus	0.069 (0.030, 0.108)	<0.001
	Ischemic heart disease	−0.033 (−0.100, 0.034)	0.340
	Cerebrovascular disease	0.022 (−0.065, 0.108)	0.624
	Liver disease	−0.036 (−0.117, 0.045)	0.386
	Cancer	0.414 (0.381, 0.448)	<0.001
Surgery time (min)		0.002 (0.001, 0.002)	<0.001
Propofol based intravenous anaesthesia		−0.155 (−0.191, −0.119)	<0.001
Intraoperative magnesium sulphate infusion		−0.223 (−0.269, −0.178)	<0.001
Years of surgery	2013–2014 (vs 2010–2012)	0.151 (0.116, 0.186)	<0.001
	2015–2016 (vs 2010–2012)	0.211 (0.179, 0.242)	<0.001
**Preoperative eGFR, mL min** ^− **1**^ **1.73 m** ^− **2**^			
	eGFR: ≥90	1	
	eGFR: 60–90	0.018 (−0.011, 0.047)	0.215
	eGFR: 30–60	−0.046 (−0.118, 0.027)	0.216
	eGFR: <30	−0.176 (−0.358, 0.005)	0.057

Variables with *P* < 0.1 (bold) and eGFR are included in the multivariable linear regression analysis in Table 3.

Preoperative eGFR^a^ (mL min^−1^ 1.73 m^−2^): 186 × (Creatinine)^−1.154^ × (Age)^−0.203^ × (0.742, if female).

ASA, American Society of Anesthesiologists; eGFR, estimated glomerular filtration rate.

**Table 3:** Multivariate generalized linear regression analysis for morphine equivalent (mg) consumption on postoperative day 0–3 after major laparoscopic abdominal surgery from 2010–2016.

		Multivariable model	
Variables		Exp. Coefficient (95% CI)	*P value*
Sex	Male (vs female)	0.351 (0.323, 0.379)	<0.001
Body Mass Index, kg m^−2^		0.019 (0.015, 0.023)	<0.001
ASA class	2 (vs 1)	−0.026 (−0.059, 0.008)	0.136
	3 (vs 1)	−0.046 (−0.126, 0.034)	0.261
Diabetes mellitus		0.010 (−0.032, 0.052)	0.646
Hypertension		0.003 (−0.034, 0.039)	0.885
Cancer		0.362 (0.329, 0.395)	<0.001
Surgery time (min)		0.001 (0.001, 0.001)	<0.001
Propofol based intravenous anaesthesia		−0.135 (−0.182, −0.088)	<0.001
Intraoperative magnesium sulphate infusion		−0.084 (−0.144, −0.024)	0.006
**Years of surgery**			
	2013–2014 (vs 2010–2012)	0.125 (0.090, 0.161)	<0.001
	2015–2016 (vs 2010–2012)	0.168 (0.136, 0.201)	<0.001
**Preoperative eGFR, mL min** ^− **1**^ **1.73 m** ^− **2**^			
	eGFR: ≥90	0	
	eGFR: 60–90	−0.022 (−0.051, 0.008)	0.145
	eGFR: 30–60	−0.046 (−0.120, 0.028)	0.222
	eGFR: <30	−0.035 (−0.064, −0.005)	0.023

Preoperative eGFR^a^ (mL min^−1^ 1.73 m^−2^): 186 × (Creatinine)^−1.154^ × (Age)^−0.203^ × (0.742, if female).

Goodness of fit, Pearson Chi-Square test, Value: 1991.1 (value/df = 0.309).

ASA, American Society of Anesthesiologists; eGFR, estimated Glomerular Filtration Rate.

Additionally, male sex (exp. coefficient: 0.323, 0.379; *P* < 0.001), body mass index (kg m^−2^) (exp. coefficient: 0.019, 95% CI: 0.015, 0.023; *P* < 0.001), cancer (exp. coefficient: 0.362, 95% CI: 0.329, 0.395; *P* < 0.001), surgery time (min) (exp. coefficient: 0.001; 95% CI: 0.001, 0.001; *P* < 0.001), propofol-based TIVA (exp. coefficient: −0.135, 95% CI: −0.182, −0.088; *P* < 0.001), intraoperative magnesium sulphate infusion (exp. coefficient: −0.084, 95% CI: −0.144, −0.024; *P* = 0.006) were significantly associated with morphine equivalent consumption during POD 0–3.

### Pain scores and adjuvant analgesic consumption during the first three PODs

Table 4 shows the results of the multivariate generalized linear regression analysis of Numeric Rating Scale (NRS) pain scores at rest, as well as the consumption of adjuvant analgesics (acetaminophen and ketorolac) on PODs 0 to 3. Preoperative eGFR was not significantly associated with NRS pain scores at rest, on PODs 0, 1, 2, or 3 (all *P* > 0.05). Among the adjuvant analgesics, acetaminophen consumption during the first three PODs was not significantly associated with preoperative eGFR (*P* > 0.05); however, ketorolac consumption during the first three PODs was 33.8% lower in the group with an eGFR of 30–60 mL min^−1^ 1.73 m^−2^ group, compared with the group with a preoperative eGFR ≥ 90 (exp. coefficient: −0.338, 95% CI: −0.596, −0.080; *P* = 0.010).

**Table 4:** Multivariate generalized linear regression for Numeric Rating Scale (NRS) pain scores at rest on postoperative day (POD) 0–3 and adjuvant analgesic use after major laparoscopic abdominal surgery from 2010–2016.

Variables	Exp. Coefficient (95% CI)	*P*-value*
**NRS pain score on POD 0 (model 1)**		
eGFR: ≥90 mL min^−1^ 1.73 m^−2^	0	
eGFR: 60–90 mL min^−1^ 1.73 m^−2^	−0.002 (−0.018, 0.015)	0.824
eGFR: 30–60 mL min^−1^ 1.73 m^−2^	−0.031 (−0.073, 0.012)	0.160
eGFR: <30 mL min^−1^ 1.73 m^−2^	−0.009 (−0.118, 0.100)	0.876
**NRS pain score on POD 1 (model 2)**		
eGFR: ≥90 mL min^−1^ 1.73 m^−2^	0	
eGFR: 60–90 mL min^−1^ 1.73 m^−2^	0.001 (−0.013, 0.015)	0.903
eGFR: 30–60 mL min^−1^ 1.73 m^−2^	−0.023 (−0.059, 0.012)	0.197
eGFR: <30 mL min^−1^ 1.73 m^−2^	−0.019 (−0.109, 0.072)	0.685
**NRS pain score on POD 2 (model 3)**		
eGFR: ≥90 mL min^−1^ 1.73 m^−2^	0	
eGFR: 60–90 mL min^−1^ 1.73 m^−2^	0.006 (−0.010, 0.021)	0.482
eGFR: 30–60 mL min^−1^ 1.73 m^−2^	0.027 (−0.012, 0.067)	0.180
eGFR: <30 mL min^−1^ 1.73 m^−2^	−0.065 (−0.169, 0.039)	0.223
**NRS pain score on POD 3 (model 4)**		
eGFR: ≥90 mL min^−1^ 1.73 m^−2^	0	
eGFR: 60–90 mL min^−1^ 1.73 m^−2^	0.001 (−0.016, 0.018)	0.921
eGFR: 30–60 mL min^−1^ 1.73 m^−2^	0.004 (−0.040, 0.048)	0.861
eGFR: <30 mL min^−1^ 1.73 m^−2^	−0.045 (−0.159, 0.069)	0.436
**Acetaminophen (mg) use in POD 0–3 (model 5)**		
eGFR: ≥90 mL min^−1^ 1.73 m^−2^	0	
eGFR: 60–90 mL min^−1^ 1.73 m^−2^	−0.021 (−0.084, 0.041)	0.508
eGFR: 30–60 mL min^−1^ 1.73 m^−2^	−0.062 (−0.209, 0.084)	0.404
eGFR: <30 mL min^−1^ 1.73 m^−2^	0.122 (−0.158, 0.402)	0.392
**Ketorolac use (mg) in POD 0–3 (model 6)**		
eGFR: ≥90 mL min^−1^ 1.73 m^−2^	0	
eGFR: 60–90 mL min^−1^ 1.73 m^−2^	−0.084 (−0.174, 0.005)	0.065
eGFR: 30–60 mL min^−1^ 1.73 m^−2^	−0.338 (−0.596, −0.080)	0.010
eGFR: <30 mL min^−1^ 1.73 m^−2^	−0.044 (−0.892, 0.804)	0.920

*P*-value*: Multivariate model for six dependent variables (NRS at rest on POD 0, 1, 2 and 3, Acetaminophen and ketorolac use in POD 0–3). Covariates of *P* < 0.2 were in univariate generalized linear regression analysis used in each multivariate generalized linear regression models.

Goodness of fit, Pearson Chi-Square test, value: 408.2, (value/df = 0.071) in model 1, value: 336.4 (value/df = 0.057) in model 2, value: 425.1 (value/df = 0.072) in model 3, value: 466.4 (value/df = 0.086) in model 4, value: 482.0 (value/df = 0.351) in model 5, and value: 2620.1 (value/df = 1.420).

Preoperative eGFR (mL min^−1^ 1.73 m^−2^): 186 × (Creatinine)^−1.154^ × (Age)^−0.203^ × (0.742, if female)

CI, Confidence Interval; eGFR, estimated Glomerular Filtration Rate.

## Discussion

This study showed that opioid consumption was significantly lower in patients with stage 4 or 5 CKD (eGFR < 30 mL min^−1^ 1.73 m^−2^) than in those with normal kidney function (eGFR ≥ 90 mL min^−1^ 1.73 m^−2^) during the first three days after major laparoscopic surgery, while it was not significantly associated with mild to moderate CKD (eGFR 30–90 mL min^−1^ 1.73 m^−2^). In addition, there were no significant differences in NRS pain scores at rest between the patients with CKD and those with normal kidney function during the first three days after major laparoscopic surgery. As a result, this study suggests that preoperative kidney function status did not affect pain outcomes after major laparoscopic surgery, except in severe CKD patients with eGFR < 30 mL min^−1^ 1.73 m^−2^. Furthermore, considering that the difference in morphine equivalent consumption between severe CKD patients with eGFR < 30 mL min^−1^ 1.73 m^−2^ and patients with normal kidney function (eGFR ≥ 90 mL min^−1^ 1.73 m^−2^) was just 3.5%, the clinical significance is questionable.

A recent study by Binhas *et al*. indicated that there was considerable variability in postoperative pain management for patients, in all the stages of CKD^12^. Moreover, Binhas *et al*. also reported that morphine is favoured even in patients with end stage renal disease^12^. However, the study of Binhas *et al*. evaluated the prescribing patterns of physicians in a single survey, therefore, the pain outcomes such as the opioid dosage or pain scores were not evaluated with respect to the eGFR status of the CKD patients. Hence, we divided patients based on their preoperative eGFR status into four categories (≥90, 60–90, 30–60, and < 30 mL min^−1^ 1.73 m^−2^), and analysed their opioid consumption during POD 0–3. Our findings are novel, because no study has evaluated the effect of preoperative CKD stage on the postoperative pain outcome in detail.

Even though the morphine equivalent consumption in our study was 3.5% lower in the patients with severe CKD than in those with normal kidney function, there was no significant difference in postoperative NRS pain scores at rest during the first three PODs between the two groups. There are two reasons that may explain this similarity in pain scores between the groups. First, it is possible that the serum opioid concentration was maintained at a similar level between patients with CKD and those with normal kidney function, regardless of dose reduction, due to delayed excretion of opioids in CKD patients. Moreover, the most commonly used opioid in this study was intravenous (IV) patient-controlled analgesia (PCA) fentanyl and the elimination of fentanyl can be delayed in CKD patients. Because the metabolites of fentanyl are known to be inactive and nontoxic^13^, it is possible that even a reduced dose of fentanyl may have resulted in prolonged analgesic effects in CKD patients. Second, all patients received an IV PCA for pain control, which enabled them to titrate their postoperative pain^14^. Thus, an opioid dose reduction in CKD patients was unlikely to result in insufficient pain control.

One notable finding of this study pertains to the appropriate dose reduction of IV fentanyl for CKD patients. According to a 2009 guideline, the dose of most opioids, including IV fentanyl, should be reduced by 25% for patients with an eGFR between 10 and 50^15^. However, a recently updated 2017 guideline reported that CKD patients do not show a significant serum accumulation of IV fentanyl^16^, suggesting that it is not necessary to reduce the dose of IV fentanyl for opioid-naïve CKD patients. The protocol at the SNUBH renders a reduction of the IV PCA fentanyl dose for CKD patients by about 25% to 50%, according to the patient’s eGFR. However, because patients were able to self-titrate and receive other opioid analgesics, the morphine equivalent consumption in severe CKD patients was only 3.5% lower than in patients with normal preoperative kidney function in the present study. Thus, our results suggest that it is not necessary to reduce the IV PCA fentanyl dose by more than 25% for CKD patients.

Another notable finding of this study is that we identified methods that can reduce postoperative pain in CKD patients, including the use of propofol-based TIVA. For several years, the SNUBH has used either an intraoperative magnesium sulphate infusion^17^ or propofol-based TIVA^18^ for laparoscopic surgeries. This study showed that intraoperative magnesium sulphate infusion resulted in a significantly lower morphine equivalent consumption (about 8.4%) and propofol-based TIVA resulted in a significantly lower morphine equivalent consumption (about 13.5%) during the first three PODs, which is in line with the findings of previous studies^19–21^. This suggests that propofol-based TIVA or magnesium sulphate infusion should be routinely considered for CKD patients. Additionally, surgery time or cancer were associated with morphine equivalent consumption during POD 0–3 in this study. This can be explained by an assumption that more complex and longer duration of surgical procedures are associated with more severe pain than simple surgical procedures. Preoperative BMI is another interesting factor associated with postoperative pain in this study. There were some reports that preoperative BMI was not associated with postoperative pain^22,23^; however, according to our study findings, there was positive association between preoperative BMI and morphine equivalent consumption during POD 0–3. At our institution, physicians did not tend to prescribe additional opioids in patients with lower BMI, which affected the results of this study. Therefore, further study is needed to confirm the association between BMI and postoperative pain outcomes at our institution.

This study has a few limitations. First, there is a possibility of selection bias due to the nature of a retrospective cohort design. In order to minimize the risk of selection bias, we used a medical record technician who was blinded to the purpose of this study. Second, this study was performed at a single centre, which may limit the generalizability of these findings. Third, most of the opioid analgesia used in this study was IV PCA fentanyl and the use of additional opioids (e.g., tramadol and oxycodone) necessitated the application of a standard conversion ratio, which may have reduced the accuracy of these results. Fourth, the patients’ pain scores at rest were assessed by many different nurses over a period of seven years, which makes it difficult to ensure the accuracy of these scores. In addition, this study did not evaluate NRS pain scores at movement during POD 0–3, which limits the results of this study. Finally, we only focused on laparoscopic abdominal surgery rather than other surgeries, such as laparotomy, thoracotomy, or amputation, which could induce higher postoperative pain than laparoscopic surgery. Therefore, the results of this study might be restricted in its application to other surgeries.

In conclusion, our results suggest that patients with mild to moderate CKD (stage 2–3) did not require reduction of opioid analgesics during POD 0–3, compared to patients with normal preoperative kidney function. Only patients with severe CKD (stage ≥ 4) might require a slight reduction of opioid analgesics.

## Methods

This retrospective cohort study was approved by the institutional review board of the SNUBH (approval number: B-1803/459-105; approval date: 2018.03.12). The requirement for obtaining informed consents was waived due to the retrospective study design, and this manuscript adheres to the applicable STROBE guidelines. The medical record technician who collected patients’ demographic, clinical, and surgery-related data was blinded to the purpose of this study.

### Patients

The medical records of patients aged 20 years or older who underwent elective, major laparoscopic surgery at the SNUBH between January 1, 2010 and December 31, 2016 were analysed. Major laparoscopic surgery was defined as any laparoscopic surgical procedure that lasted for more than 2 hours and involved resection of an intraperitoneal organ. Simple appendectomy and cholecystectomy were excluded, even if the duration of surgery was longer than 2 hours. The exclusion criteria also included emergency surgery, single-port laparoscopy (which is known to cause less pain)^24^, discharge before POD 3, additional surgery during the first three PODs, intraoperative conversion to open laparotomy, continuous infusion of remifentanil or dexmedetomidine during the first three PODs, preoperative chronic opioid usage, and incomplete or missing medical records.

During the study period, experienced surgical teams at the SNUBH proficiently performed laparoscopic resections of major intraperitoneal organs (liver, stomach, colon and rectum, pancreas). Most patients received either balanced anaesthesia with desflurane and remifentanil or total intravenous anaesthesia (TIVA) with propofol and remifentanil.

### Analgesia during the first three days after major laparoscopic surgery

Most patients received IV PCA during the first 2 PODs. Most commonly, the IV PCA contained 8 to 12 µg/mL of fentanyl in normal saline. The background infusion rate (0.8–1.2 mL/h) and bolus dose (0.5–1.5 mL) were adjusted based on the patients’ underlying disease and kidney function. In general, the background infusion rate and bolus dose were reduced by 25% to 50% in patients with low baseline kidney function. After the IV PCA was discontinued, most patients were prescribed an additional opioid analgesic (e.g., oxycodone, morphine, or fentanyl) based on the physician’s judgment or patient request. Adjuvant analgesics, such as acetaminophen and ketorolac, were also used for postoperative pain control. In general, surgeons at the SNUBH did not perform local anaesthetic infiltration of the wound site during the study period.

### Preoperative kidney function

A serum creatinine (mg dL^−1^) test is performed within one month of surgery as part of the routine preoperative laboratory screening for all elective surgical procedures at the SNUBH. We computed each patient’s preoperative eGFR using this baseline serum creatinine value and the Modification of Diet in Renal Disease Formula^25^: eGFR (mL min^−1^ 1.73 m^−2^) = 186 × (preoperative serum creatinine)^−1.154^ × (age)^−0.203^ × (0.742 if female).

### Pain outcomes during the first three days after major laparoscopic surgery

Patients’ pain was regularly assessed using the NRS (0–10; 0: no pain, 10: worst pain imaginable) at rest by registered nurses at 4- to 6-hour intervals and at least 5 times a day. The mean NRS pain scores at rest were computed for PODs 0, 1, 2, and 3. The total opioid consumption, including IV PCA and additional opioid analgesics used on PODs 0 to 3, were computed using the oral morphine equivalent consumption and summed. A standard conversion ratio was used for the conversion and calculation^26^ (Supplementary Table 1). Data were also collected on oral acetaminophen and IV ketorolac consumption during the first three PODs.

### Endpoints

The primary endpoint measured in this study was the morphine equivalent consumption during POD 0–3 according to the preoperative kidney function status. The secondary endpoints measured in this study were NRS pain scores and adjuvant analgesic consumption during the first three days after major laparoscopic surgery according to the preoperative kidney function status. We classified the preoperative kidney function into four categories (eGFR ≥ 90, 60–90, 30–60, <30 mL min^−1^ 1.73 m^−2^).

### Statistical analysis

Patients’ baseline characteristics are presented as number and percentage or mean value and SD. We examined the normality of distribution in dependent variables, such as morphine equivalent consumption, NRS at rest on POD 0–3, acetaminophen consumption, and ketorolac consumption, using the Kolmogorov-Smirnov test. After confirming that these variables did not follow a normal distribution, we decided to perform generalized linear regression analysis. First, we investigated variables associated with morphine equivalent consumption on PODs 0 to 3 using univariate generalized linear regression analysis. In this generalized linear regression model, a gamma distribution was assumed for the dependent variable (morphine equivalent consumption), and the log link function was used. The variables with *P* < 0.2 in the univariate generalized linear regression model were selected and included in the multivariate generalized linear regression analysis along with the preoperative eGFR groups (eGFR ≥ 90, 60–90, 30–60, <30 mL min^−1^ 1.73 m^−2^). For secondary endpoints, we also performed the same multivariate generalized linear regression analysis with six postoperative outcomes (NRS pain score at rest on PODs 0 to 3 and use of acetaminophen and/or ketorolac on PODs 0 to 3) as the dependent variables. Results are presented as exp. regression coefficient and 95% CI, and there was no significant multi-collinearity between variables in any of the multivariate generalized linear regression models (all variance inflation factors <2.5). Additionally, Pearson chi-square statistics were used to test goodness of fit of multivariable models. All statistical analyses were performed using SPSS software (version 24.0; IBM Corp, Chicago, IL), and statistical significance was set at *P* < 0.05. Considering that the mean (SD) morphine equivalent consumption during POD 0–3 was 516.6 (303.3) mg in this study, 143 patients were required in each group to detect 100 mg of difference in the morphine equivalent consumption during POD 0–3 between patients with normal kidney function (eGFR ≥ 90 mL min^−1^ 1.73 m^−2^) and those with CKD (eGFR < 90 mL min^−1^ 1.73 m^−2^) with a 0.05 chance of type 1 error and 80% power.

## Supplementary information

Supplmentary table 1. Equianalgesic opioid conversion table1

## Data Availability

The datasets used and/or analysed during the current study are available from the corresponding author upon reasonable request.
