# Supplementary material for: Analgesia after major laparoscopic surgery in patients with chronic kidney disease: A retrospective cohort study
Source: Sci Rep. 2019 Mar 8;9:3939. doi: 10.1038/s41598-019-40627-1 (PMC6408425; doi:10.1038/s41598-019-40627-1)
Supplement: Supplementary file 1 — Supplmentary table 1. Equianalgesic opioid conversion table1 [file 41598_2019_40627_MOESM1_ESM.docx]

**Analgesia after major laparoscopic surgery in patients with chronic kidney disease: A retrospective cohort study**

^1^Hey-ran Choi, MD, PhD, ^2^Tak Kyu Oh, MD, ^2^Jinhee Kim, MD, PhD, and ^2^Young-Tae Jeon, MD, PhD

^1^Department of Anesthesiology and Pain Medicine, Inje University Seoul Paik Hospital

^2^Department of Anesthesiology and Pain Medicine, Seoul National University Bundang Hospital

**Correspondence and reprint requests:** Tak Kyu Oh, MD

Department of Anesthesiology and Pain Medicine

Seoul National University Bundang Hospital

Gumi-ro 173 Beon-gil, Bundang-gu, Seongnam 13620, Korea

Tel: 82-31-787-7499

Fax: 82-31-787-4063

Email: [airohtak@hotmail.com](mailto:airohtak@hotmail.com)

**Running title**: Chronic kidney disease and opioid analgesia

**Supplmentary table 1. Equianalgesic opioid conversion table**

| Opioid | Administration route | Dose equivalent to 10 mg of oral morphine (mg) |
| --- | --- | --- |
| Morphine | Oral | 10 |
| Morphine | Intravenous | 3.3 |
| Hydromorphone | Oral | 2 |
| Fentanyl | Intravenous | 0.03 |
| Oxycodone | Oral | 7 |
| Codeine | Oral | 80 |
| Tramadol | Oral | 40 |
